# Con: are we ready to translate Alzheimer’s disease-modifying therapies to people with down syndrome?

**DOI:** 10.1186/s13195-014-0061-6

**Published:** 2014-09-12

**Authors:** Elizabeth Head, Frederick A Schmitt

**Affiliations:** 1 Department of Pharmacology and Nutritional Sciences, Sanders-Brown Center on Aging, University of Kentucky, 203 Sanders-Brown Building, 800 S. Limestone Street, Lexington 40536-0230, KY, USA; 2 Department of Neurology, Sanders-Brown Center on Aging, University of Kentucky, 203 Sanders-Brown Building, 800 S. Limestone Street, Lexington 40536-0230, KY, USA

## Abstract

**Background:**

Adults with Down syndrome develop Alzheimer’s disease neuropathology in an age-dependent manner. This unique feature provides an opportunity to test interventions targeted for prevention of Alzheimer’s disease neuropathology and dementia in Down syndrome.

**Discussion:**

In considering clinical trial designs, however, there are several challenges that we believe will be critical to examine further. These include: accuracy in dementia, mild cognitive impairment and preclinical Alzheimer’s disease diagnoses in Down syndrome; clinical trial outcome measures appropriate for individuals with Down syndrome; *in vivo* imaging outcome measures (and practical considerations); and contributions of medical co-morbidities to disease progression. Also, when studies are designed, the molecular target may appear to be obvious (for example, targeting beta-amyloid pathology), but chromosome 21 has over 200 additional genes that could influence both positive and negative clinical trial outcomes.

**Summary:**

Observational longitudinal studies of aging in Down syndrome will be critically important as there is a need to establish sensitive clinical outcome measures and understand the consequences of gene overexpression in relation to specific interventions.

## Introduction

Aging people with Down syndrome (DS) are at increased risk for cognitive decline, dementia, and Alzheimer’s disease (AD). In DS adults ranging from 40 to 49 years of age, up to 55% may be demented, and between 50 and 59 years, prevalence ranges from 4% to 55% [1],[2]. By the age of 40 years, virtually all DS adults have sufficient senile plaques - containing the beta-amyloid (Aβ) peptide - and neurofibrillary tangles for a neuropathological diagnosis of AD [3]. Aβ pathology, specifically, can be observed in diffuse plaques in patients as young as 30 years of age. The early age of onset of Aβ and the development of AD in DS is thought to reflect increased expression of the amyloid precursor protein (APP) on chromosome 21. Interestingly, there is dissociation between the age of onset of AD neuropathology (approximately 40 years) and age of onset of dementia (approximately 50 years). Although not all adults with DS become demented (despite the presence of AD neuropathology [2],[4]), the age dependency of the development of AD neuropathology in DS provides a unique opportunity to develop preventative interventions that can be beneficial in delaying or preventing dementia.

## Discussion

### Diagnosing dementia in down syndrome and clinical trial outcome measures

Systematic diagnostic criteria for mild cognitive impairment or dementia in DS have not been clearly established [5]-[7]. Similarly, in DS, it is not entirely clear what clinical outcome measures might be most sensitive to prevention or treatment approaches as multiple studies nationally and internationally have used different clinical outcomes to detect developmental changes in DS along with treatment benefits. It is critical to identify outcome measures that can be used across clinical trials (along with a broad age range), and they will be substantially different from those used in sporadic AD clinical trials. Indeed, concerns have been raised in regard to the selection of the neuropsychological measures for sporadic AD clinical trials that may be not as sensitive to treatment outcomes as was initially thought [8].

Clinical trials in sporadic AD are relying more upon imaging and cerebrospinal fluid (CSF) approaches to both identify appropriate persons for inclusion in studies and to potentially monitor both treatment benefits and adverse effects [8]. There are several structural imaging studies in DS with age in the literature (reviewed in [9]) but fewer studies using amyloid positron emission tomography (PET) imaging ligands [10]. Given that the age of onset of clinical dementia in DS is over 50 years of age and can be variable (including some individuals who do not apparently develop dementia), further longitudinal PET studies would be critical to establish its use as a clinical outcome measure independent of cognitive measures.

### Practical considerations in clinical trials for down syndrome

The practical considerations of initiating a clinical trial for people with DS should be discussed. Identifying and recruiting DS adults can be a slow process, although the new DS-Connect registry [11] will significantly enhance this effort. Retention may also be challenging as various co-morbidities that develop with age in DS will need to be managed. An excellent example comes from a 2-year antioxidant trial in demented adults with DS in which clinical decline was influenced by the presence of seizures [12]. Although obtaining blood samples from persons with DS is not without challenges, this can often be readily accomplished. However, given the impact of DS physiology, intellectual disability, and associated anxiety over more invasive procedures, CSF samples are likely to be more difficult to acquire. Further, more invasive techniques are likely to produce added concerns for participants, family, and caregivers that would likely also lead to increased institutional review board concerns over consent (family and/or caregiver) versus assent by the participant. Similarly, our own experience suggests that imaging studies in DS are associated with additional challenges, including motion artifact, unwillingness to enter the scanner, and an inability to tolerate the prone position for great lengths of time [13].

### Selection of the intervention approach

Which intervention is appropriate? One of the most consistent features of aging in DS is the progressive accumulation of Aβ in patients over the age of 30 years. Targeting the elimination or reduction of Aβ is a very reasonable goal for adults with DS as a prevention approach. Will preventing or slowing Aβ deposition in adults with DS prevent cognitive decline and dementia as they age? For this discussion, we will focus on Aβ-targeted therapies (many different prevention approaches that are vaccine-based or pharmacological could be considered) and challenges for clinical trials for adults with DS that are, we suggest, unique to this vulnerable cohort.

It is important to remember that chromosome 21 contains over 200 genes, affecting multiple protein pathways and domains that have not been fully explored in relation to aging and that could significantly, and possibly adversely, affect clinical trial outcomes. For example, neuroinflammation is a feature of the DS aging brain, but studies are still limited (reviewed in [14]). Interestingly, approximately a dozen genes on chromosome 21 are directly related to inflammation. This suggests a disruption in the inflammatory milieu in DS that could directly modulate responses to interventions that include immunotherapy or anti-inflammatories. Targeting APP overexpression by beta-secretase (beta-amyloid cleaving enzyme, or BACE) inhibitors, which are still considered a possible therapy and are currently in clinical trials in AD [15], is also promising. However, BACE1 has multiple neuronal substrates that can include several genes overexpressed in DS (for example, the neuroinflammatory gene *IFNAR2*), not to mention that BACE2 (a homologous protein) is on chromosome 21, which may complicate the interpretation of the outcomes.

## Summary

Careful studies establishing longitudinal aging outcomes in non-treated individuals with DS will greatly inform prevention studies similar to the planned Down Syndrome Biomarker Initiative study [16]. Overall, establishing appropriate clinical outcome measures and considering possible complications related to the fact that multiple genes are overexpressed in DS that can affect inflammation, oxidative stress, mitochondrial dysfunction, and other mechanisms are important to consider in terms of both efficacy and adverse events.

## Abbreviations

AD: Alzheimer’s disease

APP: Amyloid precursor protein

Aβ: Beta-amyloid

BACE: Beta-amyloid cleaving enzyme

CSF: Cerebrospinal fluid

DS: Down syndrome

PET: Positron emission tomography

## Competing interests

The authors declare that they have no competing interests.
